# Cognitive stimulation program in mild cognitive impairment A randomized controlled trial

**DOI:** 10.1590/1980-57642020dn14-020003

**Published:** 2020

**Authors:** Isabel Gomez-Soria, Patricia Peralta-Marrupe, Fernando Plo

**Affiliations:** 1 University of Zaragoza Ringgold standard institution Zaragoza, Aragón, Spain.; 2 Royal Devon and Exeter Hospital Ringgold standard institution Barrack Road, Exeter EX2 5DW United Kingdom of Great Britain and Northern Ireland.

**Keywords:** cognitive dysfunction, aging, randomized controlled trial, occupational therapy, disfunção cognitiva, envelhecimento, estudo controlado randomizado, terapia ocupacional, estimulação cognitiva, comprometimento cognitivo leve

## Abstract

Non-pharmacological cognitive interventions in mild cognitive impairment have demonstrated promising results in preventing or delaying cognitive impairment and functional disability. Cognitive stimulation seems to improve and maintain cognitive and social activity.

**Objective:**

This study aimed to evaluate the impact of a cognitive stimulation program in mild cognitive impairment (MCI) at the cognitive level on activities of daily living (ADLs), and levels of anxiety and depression.

**Methods:**

A randomized controlled single-blind trial involving 122 non-institutionalized elderly with a MEC-35 score of 24-27 was conducted. The intervention group (n=54) received the intervention (10-week cognitive stimulation program) and was compared with a control group (n=68) that received no intervention. Follow-up assessments were conducted post-test and at 6 months post-test. The primary outcome was cognitive function determined by changes in scores on the Spanish version (MEC-35) of the Mini-Mental State Examination, while the secondary outcomes were measured by the Barthel Index, Lawton and Brody Scale, Goldberg Questionnaire (anxiety sub-scale) and the Yesavage Geriatric Depression Scale (15-item version).

**Results:**

The intervention group showed a significant improvement in cognitive function at both timepoints, post-test and 6-month follow-up. The Barthel Index was higher in the intervention group, but only on the post-test analysis. The intervention did not improve the performance of instrumental ADLs or depression or anxiety levels.

**Conclusion:**

The findings showed cognitive improvements in an elderly population with MCI in the short and medium-term and improved basic ADLs in the short term. Clinicaltrials.gov Identifier: NCT03831061.

## Resumo.

As intervenções cognitivas não farmacológicas no comprometimento cognitivo leve demonstram resultados promissores na prevenção ou retardo no comprometimento cognitivo e na incapacidade funcional. A estimulação cognitiva parece melhorar e manter a atividade cognitiva e social.

**Objetivo:**

Nosso objetivo foi o de avaliar um programa de estimulação cognitiva sobre o comprometimento cognitivo leve no nível cognitivo, nas atividades da vida diária, níveis de ansiedade e depressão.

**Métodos:**

Um estudo randomizado, controlado e cego, foi implementado em 122 idosos não institucionalizados com escore 24-27 na versão em espanhol do Mini Exame do Estado Mental (MEC-35). O grupo de intervenção (n=54) recebeu a intervenção (programa de estimulação cognitiva de 10 semanas) e foi comparado com um grupo de controle (n=68) que não recebeu nenhuma intervenção. Avaliações de acompanhamento foram realizadas pós-teste e 6 meses pós-teste. O desfecho primário foi a função cognitiva determinada pelas alterações nos escores do MEC-35 e os desfechos secundários foram medidos pelo Índice de Barthel, Escala de Lawton e Brody, Escala de Lawton e Brody, Questionário Goldberg (subescala de ansiedade) ) e a Escala de Depressão Geriátrica de Yesavage (versão de 15 itens).

**Resultados:**

O grupo intervenção apresentou uma melhora significativa na função cognitiva nas duas medidas de tempo, pós-teste e 6 meses de acompanhamento. O Índice de Barthel foi encontrado mais alto no grupo de intervenção, mas apenas na análise pós-teste. A intervenção não melhorou o desempenho de atividades instrumentais da vida diária e níveis de depressão ou ansiedade.

**Conclusão:**

Os achados mostram melhorias cognitivas no comprometimento cognitivo leve da população idosa em curto e médio prazo e melhoraram as atividades básicas da vida diária em curto prazo. Identificador do Clinicaltrials.gov: NCT03831061.

We are experiencing a huge demographic shift, with an increase in the elderly population and prevalence of ageing-related diseases.^1^ Mild cognitive impairment (MCI) reflects a level of cognitive functioning between ageing and dementia. MCI is an especially big challenge and the development of non-pharmacological interventions is critically needed.^2^ MCI prevalence is increasing with age and has an incidence of between 21.5 and 71.3 per 1,000 population/year.^3^ The annual rate of progression to dementia ranges from 8% to 15%.^4^

The MCI concept has led to debate regarding the value of non-pharmacologic interventions.^5^ Non-pharmacological cognitive interventions could be key in preventing or delaying cognitive impairment and functional disability.^6^ Clare & Woods (2004)^7^ describe three different types of cognitive intervention: cognitive training, cognitive rehabilitation and cognitive stimulation. Cognitive training refers to guided standard tasks to develop cognitive function. Cognitive rehabilitation focuses on the improvement of some cognitive goals. Finally, cognitive stimulation includes participation in cognitive activities, mainly in groups, designed to improve and maintain social and cognitive activity. Cognitive stimulation includes activities such as orientation, reminiscence, memorization, association and leisure activities. These three types of intervention are based on unimodal interventions (focusing on one domain). Multimodal cognitive interventions are generally more complex interventions (encompassing physical, social or psychological components).^8^

A number of studies based on cognitive stimulation have shown improvements in cognitive function in healthy older people, elderly with MCI and with dementia.^9^^-^ 15 The findings of Alves et al.^15^ suggest that cognitive stimulation can lead to high values of experiential relevance, even in the absence of cognitive or functional improvements. The study of Schultheisz et al.^16^ supports cognitive stimulation programs as a resource for improving cognition and quality of life for the elderly. The prevention of dementia should be taken into account in health systems given the severity of this pathology.^17^

This study seeks to determine the effectiveness of a cognitive stimulation program using a randomized controlled trial (RCT). More specifically, there were three objectives: (i) to ascertain efficacy at the cognitive level using the 35-point Cognitive Mini-Exam (MEC-35); i.e. the Spanish version of Folstein’s Mini-Mental State Examination (MMSE); (ii) to measure changes in activities of daily living (ADLs) using the Barthel Index and the Brody and Lawton Scale; (iii) to examine effects on levels of anxiety using the anxiety sub-scale, Goldberg questionnaire (EADG), and on depression using the Yesavage Geriatric Depression Scale (15-item version).

## METHODS

### Design setting

A randomized controlled trial (RCT) was performed in non-institutionalized elderly people. The inclusion criteria were being over 65 years old, not being institutionalized, not having received cognitive stimulation in the last year, scoring >60 points on the Barthel Index, and presenting no deafness, no blindness, no neuropsychiatric disorders or motor difficulties, and having a MEC-35 score of between 24 and 27 points. MEC-35 scores of less than 27 denote cognitive deficits.^18^ The optimal cut-off point on the MEC-35 to establish the presence of cognitive impairment in the population over 65 years is 24 points for a low educational level and 27 points for a medium-high level.^19^ A sample size >53 in each group guaranteed that an increase of 1.5 points on the MEC-35 could be detected with a level of significance of 5% and statistical power of 80%, assuming a standard deviation ≤2.5 points and a rate of abandonment of 35%. The CONSORT standards^20^ and the Declaration of Helsinki of the World Medical Association - Ethical Principles for Medical Research in Humans 2013^21^ were observed during the study. This study was approved by the Ethical Committee of Clinical Studies of Aragón in Act No. 18/2011, under study registration number PI11/00091 and registered on ClinicalTrials.gov Identifier (NCT03831061).

### Participant selection

The participants were recruited from San José Norte-Centro Health Center in Zaragoza (Spain). For randomization, an opaque urn was used into which the participants’ file numbers were placed and an anonymous person drew the selected numbers. The first author verified the inclusion criteria of the participants. A total of 416 candidates were evaluated. Following inclusion, the 122 patients were allocated into two groups: 54 participants in the Intervention group and 68 participants in the Control group. The evaluators and the occupational therapist who performed the intervention were different.

**Figure 1:**
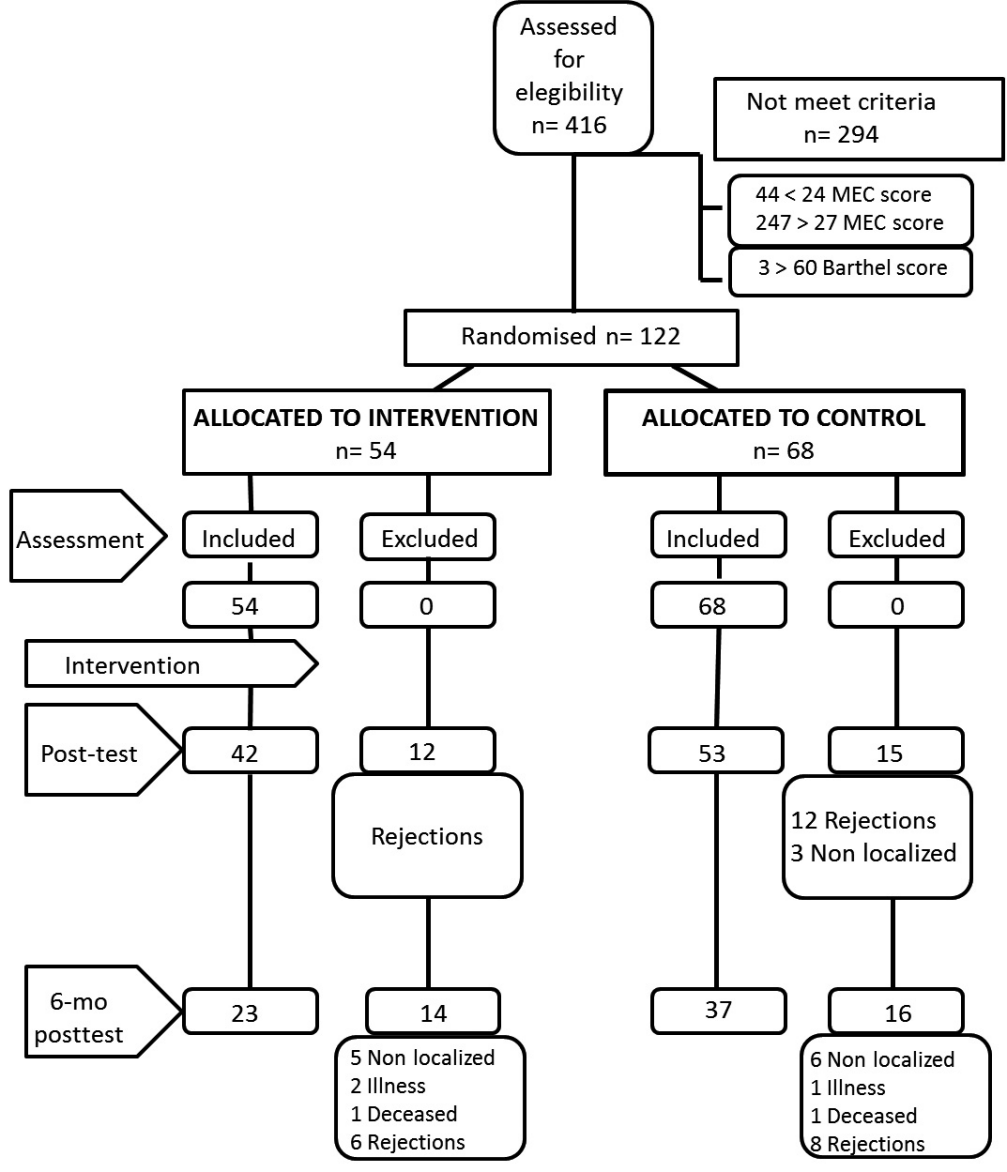
Flow chart of participation and study design.

The randomized controlled trial was single-blind, as the persons responsible for the assessments were blinded and different from those responsible for the intervention. The sample size was calculated in such a way that an increase of 1.5 points on the MEC-35 could be detected with a level of significance of 5% and a statistical power of 80%, assuming a standard deviation ≤2.5 points and a rate of abandonment of 35%. The flow of participants including the number of dropouts and their causes are shown in Figure 1. As expected, the number of dropouts was high (22.1% between pre-test and post-test). Differences in the baseline values (pre-test) of participants who stayed until the last assessment versus those who had left at some stage during follow-up were analyzed. No statistically significant differences in age or in any of the other outcome variables were found.

### Intervention

Eight occupational therapists performed the assessments (pre-test, post-test and 6-month post-test) and were all blinded. Two trained occupational therapists performed the intervention. The intervention was carried out at the Foundation La Caridad, Zaragoza (Spain) in two subgroups of 27 participants each using the red notebook of mental activation.^22^ The difficulty of the exercises was adapted to take into account cognitive level, interests and gender, as per the Spector et al.^23^ programme.

**Figure 2:**
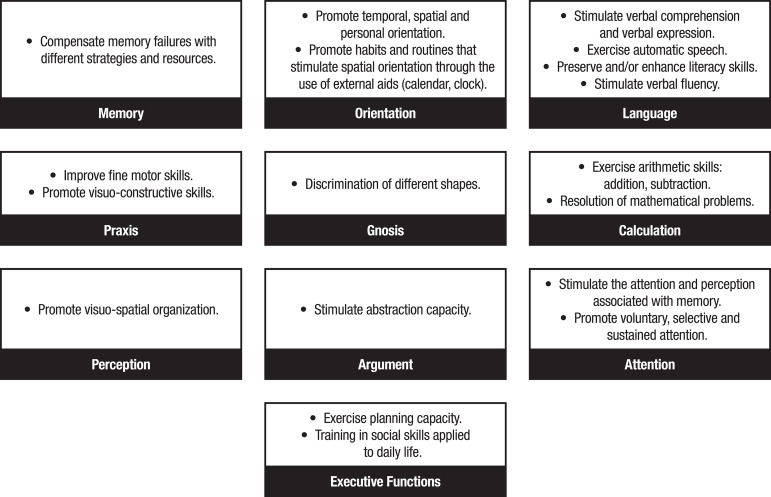
Development objectives following the red mental activation notebook guidelines.

**Figure 3:**
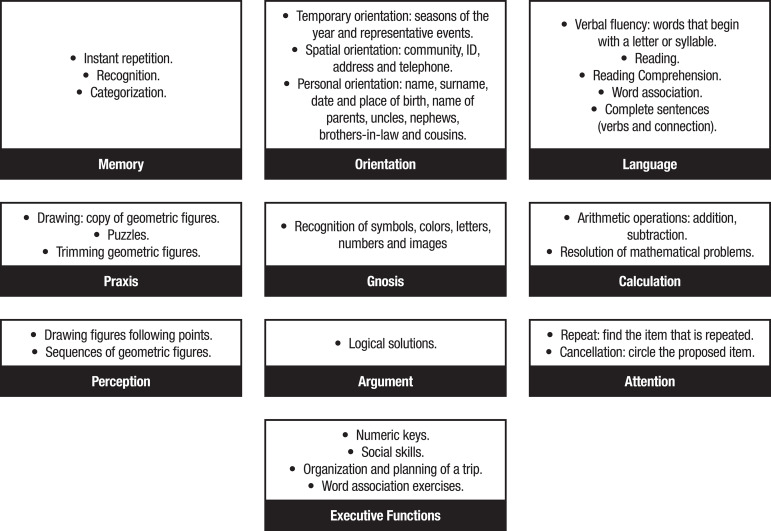
Cognitive stimulation exercises used based on red mental activation notebook guidelines.

The intervention consisted of 10 sessions of 45 min/week for 10 weeks. Each session included four parts: (a) Reality orientation: questions about date, time and place, using calendars, clock and posters indicating the place and address where the participants were situated; (b) Explanation of the cognitive aspect that was going to be focused on in each session; with alternatives including: 1) “memory” (changes withj aging, types of memory, strategies such as association and categorization); 2) “orientation” (temporary, spatial and personal); 3) “language”; 4) “praxis” (ideomotor, ideational and constructive); 5) “gnosis”; 6) “calculation”; 7) “perception”; 8) “reasoning”; 9) “visual attention”; 10) “executive functions” (planning capacity, training in social skills and association with activities of daily living); (c) Individual practical work, in which 4 exercises of the cognitive aspect corresponding to each session were performed; (d) Group correction of practical exercises. The objectives and types of cognitive stimulation exercises used in the intervention are given in Figures 2 and 3.

The conceptual framework of this intervention was formed from the framework for practice of Occupational Therapy,^24^ the cognitive model^25^ and the human occupation model of Gary Kielhofner.^26^

### Outcomes

#### Main outcome

The Mini-Mental State Examination (MEC-35) is the Spanish version of the MMSE.^27^ It is a standardized screening instrument widely used in the detection of cognitive deterioration that explores a set of cognitive functions.^19^ Scores ≤27 denote cognitive deficits. Test-retest reliability: weighted kappa = 0.667, sensitivity = 89.8%, and specificity = 83.9%.^18^ The 35-point questionnaire consists of 11 items in which 8 cognitive areas are assessed: space-temporal orientation, fixation and recent memory, attention-concentration and calculation, comprehensive and expressive language, abstract thinking and visuospatial construction.

#### Secondary outcomes

The Barthel Index (BI) assesses the level of independence for ten basic Activities of Daily Living (ADLs). Its internal consistency is 0.90; interobserver reliability, Kappa index is between 0.47 and 1.00, and intraobserver reliability Kappa index is between 0.84 and 0.97. Regarding the evaluation of internal consistency, a Cronbach’s alpha of 0.90-0.92^28^ was obtained. Maximum score on the Barthel Index is 100, where scores >60 indicate low dependence for ADLs and scores <20 demonstrate a high dependence level for ADLs.^28^

The Lawton and Brody Scale assesses the degree of autonomy for eight instrumental ADLs necessary for living independently in the community. Score ranges from 0 (dependent) to 8 (independent) points. The scale’s sensitivity is 0.57 and specificity is 0.92.^29^

The Goldberg questionnaire (EADG) consists of two sub-scales, one for anxiety and the other for depression. Each sub-scale has 9 dichotomous response items (Yes / No). An independent score is given for each scale, with one point for each affirmative answer. Goldberg et. al.^30^ proposed a cut-off point of ≥4 for the anxiety scale. In the present study, the anxiety sub-scale was used, which has overall specificity of 91% and sensitivity of 86%.

The Yesavage Geriatric Depression Scale (15-item version) evaluates depression level. The abbreviated version has 15 questions and is adequate for elderly people living in the community. It was highly correlated with the original version consisting of 30 items (r=0.84, p<0.001). The authors found that a cut-off score of 11 on the GDS yielded an 84% sensitivity rate and 95% specificity rate.^31^

Besides these outcome variables, other socio-demographic variables such as age, sex, marital status (single, married, widowed/separated) and educational level (primary, secondary) were collected using a structured interview.

### Data analysis

The IBM SPSS Statistics v. 22 software package was used for statistical analysis. In addition to the usual descriptive tools and Fisher’s exact test, Student’s *t*-test for equal means was used and, when statistically significant differences were found, effect size was calculated using Cohen’s d, providing both a point estimate and confidence interval. Analysis of covariance was used to control the effect of the sex variable on the main outcome variable, and the partial eta-squared statistic was used to report effect size. The significance level for statistical tests was 5% (p<0.05).

## RESULTS

The frequencies and proportions for the sociodemographic variables are shown in Table 1. Participant age was similar for both groups, ranging from 65 to 88 years (74.3±5.8 years in the intervention group and 75.6±6.2 years in the control group), with a higher proportion of women in both groups (87.0% in the intervention group and 69.1 in the control group). Randomization did not produce statistically significant discrepancies except for sex (p=0.029), but this discrepancy had no effect on the results for the effect of the intervention, as will be discussed later.

**Table 1:** Frequencies and percentages for sociodemographic variables.

Variables		Intervention Group (n=54)	Control Group (n=68)
Sex	Male	7 (13.0)	21 (30.9)
	Female	47 (87.0)	47 (69.1)
Marital status	Single	3 (5.6)	7 (10.3)
	Married	33 (61.1)	42 (61.8)
	Widowed. Separated	18 (33.3)	19 (27.9)
Educational Level	Primary	50 (92.6)	58 (85.3)
	Secondary	4 (7.4)	10 (14.7)

### Comparison of intervention and control groups

Means and standard deviations of the outcome variables in the three assessments (pre-test, post-test and 6-month post-test) are presented in Table 2.

**Table 2:** Mean±SD of outcome variables.

Variables	Intervention Group				Control Group		
	Pre-test n=54	Post-test n=42	6-month post-test n=28		Pre-test n=68	Post-test n=53	6-month post-test n=37
MEC-35	25.91±1.03	28.85±2.95	29.64±2.60		25.62±1.02	26.60±4.03	27.08±4.07
Barthel	95.93±7.65	96.43±6.27	95.89±8.50		95.74±6.18	94.28±7.64	93.74 ±8.71
Lawton-Brody	7.26±1.28	7.26±1.23	7.29±1.33		6.51±1.93	6.36±1.87	6.70±1.76
Goldberg	3.22± 2.29	2.89±2.34	2.61±1.90		2.78±2.55	2.94±2.31	2.85±2.28
GDS-15	2.93±2.60	2.83±2.97	2.13±1.86		3.14±2.89	3.62±3.35	3.12±3.52

Outcome variables expressed in points on respective scales. MEC-35: Mini-Examen Cognoscitivo-35 points (Spanish version of MMSE). Barthel: Barthel Index. Lawton-Brody: Lawton and Brody Scale. Goldberg: Goldberg Anxiety Sub-scale. GDS-15: Yesavage Geriatric Depression Scale, 15-item version.

On the MEC-35 scale, both groups scored 25 at baseline, demonstrating low cognitive impairment. During the course of the study, the Intervention group improved their MEC-35 score, with an increase to 29 points. Surprisingly, the Control group improved their score from 25 to 27 points (cut-off score).

Regarding both instrumental and basic ADLs, measured by the Lawton & Brody Scale and Barthel Index, respectively, participants had no dependence at study baseline. The intervention did not improve the performance of ADLs. However, the Control group showed a decline in scores during the intervention period, while the Intervention group maintained their score on the Barthel Index.

The analysis of anxiety and depression levels revealed no difference between the Intervention and Control groups. At study baseline, the Intervention group had a score of 3 on the Goldberg questionnaire, close to the cut-off point of 4. However, the intervention was able to reduce anxiety levels with score decreasing to 2.

Randomization produced no statistically significant discrepancies for any of these variables. Therefore, pre-test values were very similar for all variables, but better behavior was evident in the Intervention group on the MEC-35, both at post-test and 6-month post-test. To assess the effect of the intervention, increments over the baseline level of the outcome variables were calculated along with their differences between Intervention and Control group, both after the intervention (post-test) and after 6 months (6-month post-test), as presented in Table 3. Statistically significant differences were found in MEC-35 post-test scores (1.91 points, p=0.005) with Cohen’s d of 0.564 and 95% confidence interval (0.150, 0.975), and in MEC-35 6-month post-test scores (2.34 points, p=0.009) with d=0.764 and 95% confidence interval (0.253, 1.270). Analysis of covariance was used to control the effect of the sex variable for these increases on the MEC-35, and this analysis ruled out interaction between participant sex and the effect of the intervention on both the post-test assessment (F=0.807, p=0.371) and 6-month post-test assessment (F=1.749, p=0.191). The linear model used estimated the effect of the intervention, after controlling for sex, at 1.63 points for the post-test evaluation and at 2.06 points for the 6-month post-test follow-up. A statistically significant difference was also found in performance on the Barthel post-test (2.01 points, p=0.048) with Cohen’s d of 0.416 and 95% confidence interval (0.004, 0.826). This difference decreased to 1.12 points on the 6-month post-test assessment and was no longer statistically significant. No statistically significant difference in performance was observed on the Lawton-Brody, Goldberg or GDS-15 instruments.

**Table 3:** Mean±SD of increases in outcome variables relative to baseline levels.

Variables	Post-test					6-month post-test			
	IG n=42	CG n=53	IG-CG	p		IG n=28	CG n=37	IG-CG	P
MEC-35	2.89±2.65	0.98±3.87	1.91	0.005		3.78±2.49	1.44±3.43	2.34	0.009
Barthel	0.71±4.49	-1.30±5.08	2.01	0.048		-0.18±7.00	-1.49±7.42	1.21	0.506
Lawton-Brody	-0.05±0.44	-0.15±0.97	0.10	0.491		-0.04±0.88	0.11±1.24	-0.15	0.604
Goldberg	0.03±2.26	-0.01±2.48	0.04	0.927		-0.52±2.36	-0.12±2.42	-0.40	0.619
GDS-15	-0.12±1.91	0.13±2.73	-0.25	0.600		-0.73± 2.82	-0.39±2.64	-0.34	0.600

Outcome variables expressed in points on respective scales. MEC-35: Mini-Examen Cognoscitivo-35 points (Spanish version of MMSE). Barthel: Barthel Index. Lawton-Brody: Lawton and Brody Scale. Goldberg: Goldberg Anxiety Sub-scale. GDS-15: Yesavage Geriatric Depression Scale, 15-item version. IG-CG: difference in means between Intervention and Control Groups. p: p-value of Student's t-test of equal means.

## DISCUSSION

The aim of this study was to assess the results of a cognitive intervention program in elderly people. Our results demonstrate that the intervention may help participants’ cognitive performance and basic activities of daily living. The Intervention group showed a significant improvement in cognitive function, as measured by the MEC-35 scale, after the intervention both at post-test and 6-month follow-up. The Barthel Index was also higher in the Intervention group, but only on the post-test analysis. However, no significant differences were found for the Lawton and Brody Scale. There were no statistically significant differences between Intervention and Control groups on the Goldberg Questionnaire or the Yesavage Geriatric Depression Scale. It should be noted that the pre-test scores in both groups were relatively low, meaning that participants did not present anxiety or depression.

Both post-test and 6-month post-test results showed that the program produced positive results in the Intervention group, with statistically significant improvements in general cognitive state, as measured with the Spanish version of the MMSE (MEC-35). The effect size of the post-test analysis was Cohen’s d=0.564, a medium size according to Belleville et al..^32^ Our results are in line with some previously published studies. Llanero-Luque et al.^14^ reported a similar post-test effect size for the MEC-35 (d=0.45) after performing a cognitive stimulation program. Polito et al.^13^ reported statistically significant short-term gains on the MMSE after applying this in MCI participants living in the community, as did the study by Alves et al.^15^ of institutionalized participants with either MCI or mild/moderate dementia, both through a cognitive stimulation program.

Our medium-term effect size at the 6-month post-test on the MEC-35 was Cohen’s d=0.764, a large value according to Belleville et al.^32^. Hwang, et al.^33^ described a cognitive training program involving MCI participants, but did not report a statistically significant improvement in the medium term on the MMSE.

We found statistically significant post-test improvements in basic ADLs measured with the Barthel Index for the Intervention group, but this improvement was small and no longer significant on the 6-month post-test. A longer intervention could be required to improve physical status and ADL development in the long term.

By contrast, no statistically significant improvement was obtained in instrumental ADLs, as measured with the Lawton and Brody scale, over short or medium-terms. No statistically significant improvements in ADLs are reported by similar studies that used cognitive rehabilitation.^34^^,^ 35

As we have indicated previously, none of the groups presented depression or anxiety. Consequently, no statistically significant differences were found in levels of anxiety or depression, as measured by the Goldberg and GDS-15 scales. This corroborates other studies that also found no short-term differences after a program of cognitive stimulation on the Goldberg^12^ or GDS-15.^14^ However, Talassi et al.,^35^ in a cognitive rehabilitation program, reported statistically significant differences in short-term assessments, but employed other instruments, using the STA for anxiety level and the GDS-30 for depression level.

Therefore, our hypothesis is that the present program may improve participants’ cognitive performance and basic activities of daily living in the short term.

### Limitations

First, we could not access patients’ medical history or clinical diagnosis, and pharmacological treatments were not recorded. Second, we had a high number of dropouts, but this problem is difficult to avoid, and has occurred in other studies.^12^ Third, the therapists who performed the intervention and the participants could not be blinded.

### Future research

Most MCI studies involve small samples^35^^,^ 36 therefore studies with a larger sample of participants are necessary to be able to expand the knowledge in this field. There are few RCTs and their designs vary greatly^33^ with high heterogeneity in cognitive intervention techniques, time and duration of sessions, involving treatment of not only older people with MCI, but also patients with dementia and Alzheimer’s disease, whose results are evaluated by means of different questionnaires, and follow-up applied at different timepoints. This lack of methodological uniformity could explain the variability of results. It would be useful to implement RCTs with a multimodal intervention, a wider range of assessment instruments and more assessment periods. It would also be valuable to study ways of fostering participant adherence to the program in order to reduce dropouts.

In conclusion, people over 65 with MCI benefited from a cognitive stimulation program. This program may increase cognitive levels and delay cognitive impairment progression. We found that this program also improved basic ADLs of the participants in the short term.
